# Research hotspots and trends of exercise for sarcopenia: A bibliometric analysis

**DOI:** 10.3389/fpubh.2023.1106458

**Published:** 2023-03-08

**Authors:** Qing Xiang, Yue Hu, Jiaqi Zheng, Weilin Liu, Jing Tao

**Affiliations:** ^1^Rehabilitation Technology Innovation Center by Joint Collaboration of Ministry of Education and Fujian Province, Fujian University of Traditional Chinese Medicine, Fuzhou, China; ^2^College of Rehabilitation Medicine, Fujian University of Traditional Chinese Medicine, Fuzhou, China

**Keywords:** sarcopenia, exercise, skeletal muscle, bibliometric analysis, CiteSpace

## Abstract

Exercise is an effective method for the prevention and treatment of sarcopenia, which can improve skeletal muscle mass, strength and physical function in individuals with sarcopenia to varying degrees. Moreover, exercise has an important role in improving ability to perform daily activities and quality of life on sarcopenia. In this study, articles and review articles on exercise interventions for sarcopenia from January 2003 to July 2022 were retrieved from the Web of Science core collection. Then, the number of annual publications, journal/cited journal, country, institution, author/cited author, references and keywords were analyzed using CiteSpace 6.1.R2. A total of 5,507 publications were collected and the number of publications increasing each year. Experimental Gerontology was the most productive journal and the most cited journal was J GERONTOL A-BIOL. The United States of America was the most influential country with the largest number of publications and centrality. Maastricht University in the Netherlands is the most productive institution. The author VAN LOON LJC has the highest ranking in terms of publications and CRUZ-JENTOFT A is ranked first in terms of cited authors. The most frequently occurring keywords in the field of exercise interventions for sarcopenia are “skeletal muscle,” “exercise,” “body composition,” “strength,” and “older adult”; the keyword “elderly men” showed the strongest explosive intensity. The keywords formed 6 clusters, namely “skeletal muscle,” “muscle strength,” “heart failure,” “muscle protein synthesis,” “insulin resistance” and “high-intensity interval training.” In conclusion, this study demonstrates a new perspective on the current state of research and trends in exercise interventions for sarcopenia over the past 20 years *via* the visualization software CiteSpace. It may help researchers to identify potential collaborators and partner institutions, hotspots and research frontiers in the field of exercise interventions for sarcopenia.

## 1. Introduction

Sarcopenia is defined as age-related loss of muscle mass, plus low muscle strength, and/or low physical performance by the Asian Working Group for Sarcopenia (AWGS) (1). With the increasing aging of the global population, sarcopenia has become a common disease in the elderly, which not only increases the risk of falls, disability and death (2), but is also associated with a significantly higher risk of cardiovascular disease (3, 4). Sarcopenia seriously impair the physical health and quality of life of the elderly. Therefore, it is necessary to find and develop low-cost, easily accessible rehabilitation methods for sarcopenia. However, there are no effective therapeutic drugs for sarcopenia (5) and exercise plays an important role in its prevention and treatment. Physical activity is one of the recommendations for the management of sarcopenia, which was given by the International clinical Practice Guidelines for Sarcopenia in 2018 (6). The commonly used exercise methods include resistance exercise, aerobic exercise, combination of resistance and aerobic exercise, balance training, flexibility training and other auxiliary exercise methods. These exercises have a positive effect on increasing muscle mass and strength in patients with sarcopenia. These exercises have a positive effect on increasing muscle mass and strength in people with sarcopenia.

Therefore, it is necessary to conduct a comprehensive summary of the research about exercise interventions for sarcopenia. This study used the visualization software CiteSpace to analyze 5,507 retrieved records from January 2003 to July 2022 related to exercise interventions for sarcopenia. Based on a visual bibliometric analysis of the literature from the perspectives of the number of annual publications, countries, institutions, authors, keywords and citations, show the current status and development trend of exercise interventions for sarcopenia. The purpose of this study is to provide a reference for future research about exercise interventions for sarcopenia.

## 2. Materials and methods

### 2.1. Database selection

In this study, the Web of Science (WOS) core collection was selected as the data source. Web of Science, as a high-quality digital literature resource database, has been accepted by many researchers. Furthermore, the Web of Science Core Collection has a rigorous selection process based on law of Bradford and includes only the most important scholarly journals in each subject area. Web of Science core collection is regarded as the most suitable database for bibliometric analysis (7).

### 2.2. Search strategy

The search strategy we used was entering the search expression: Topic = (sarcopenia) AND Topic = (“exercise” OR “training” OR “sport” OR “movement” OR “motion”) in the Web of Science core collection. The scope of topic search includes the following fields: title, abstracts, author keywords, and keywords plus. At the time of retrieval, the publication date of the literature was unrestricted. The retrieval date was July 24, 2022. The literature type was selected as article and review article, and the language chose English. Finally, we obtained a total of 5,507 publications related to exercise interventions for sarcopenia.

### 2.3. Data standardization

All bibliometric records of the 5,507 retrieved literature were downloaded as “full records and cited references” from SCIE. Microsoft Excel 2021 (Microsoft Corporation, Redmond, WA, USA) and CiteSpace 6.1.R2 (Drexel University, Philadelphia, PA, USA) were used to standardize, analyze, and visualize the records.

All records need to be standardized before the bibliometric analysis. Above all, CiteSpace was used to remove the potential duplicate records. Since different names may appear for specific authors or institutions and lead to computational errors, we manually screened the names of identified authors and institutions with high publication volumes and merged the information after confirming that it came from the same author or institution.

### 2.4. Bibliometric analysis

Descriptive analyses of bibliometric indicators, including the annual number of publications, journals, countries, authors and keywords were analyzed in Microsoft Excel 2021.

In this study, CiteSpace was used to draw the knowledge maps, and the co-occurrence network maps of annual publications, journals, countries, institutions, authors, references and keywords were constructed. CiteSpace is a Java-based application, developed by Professor Chaomei Chen, which visualizes interrelationships between scientific articles according to their co-citation patterns (8, 9). The visual knowledge maps display the networks as the commonly seen types of node-and-link diagrams. Nodes in different networks can represent different elements, such as country, author, institution and keyword. The size of the node, which generally indicates the frequency of citation or appearance, and the different colors of the nodes indicate different years. The warmer the color, the more recent the year, and the cooler the color, the more distant the year. Links between nodes indicate collaboration or co-occurrence or co-referencing relationships. The purple ring represents centrality, and nodes with high centrality (>0.1) are usually considered as key points or turning points in a specific domain. The version of the software is constantly updated, and the version used in this study is 6.1.R2 (64-bit).

## 3. Results

### 3.1. Analysis of annual publications

**Figure 1:**
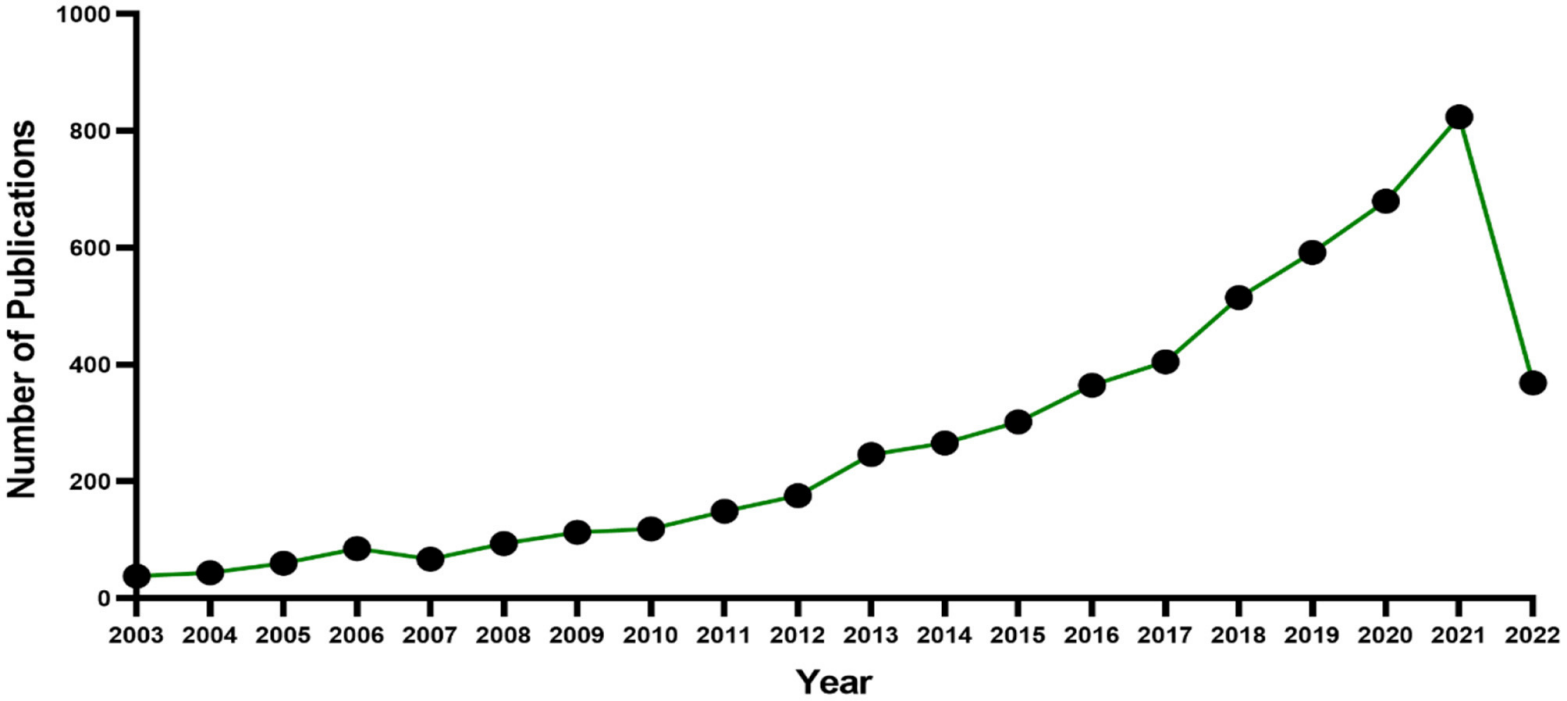
Annual number of published articles from 2003 to 2022.

There are 5,507 records related to exercise interventions for sarcopenia in the Web of Science core collection. The number of publications displayed by year is shown in Figure 1. From the figure, we can see the literature in this field was first published in 2003, and the number of publications has increased year by year. The number of publications in 2022 is only counted from January to July, but it is predicted that the number of publications in 2022 will also show an upward trend. From 2003 to 2013, the number of publications was small, but the overall trend was on the rise, indicating that the research related to exercise interventions for sarcopenia gradually attracted the attention of researchers during this period. After 2013, the number of publications increased rapidly, indicating that the research in this field has been highly valued by researchers at this stage, and exercise rehabilitation has become the focus of sarcopenia.

### 3.2. Analysis of journals and cited journals

The top five journals with the largest number of published exercise interventions for sarcopenia studies are listed in Table 1. Among these five journals, Experimental Gerontology, Journals of Gerontology Series A-Biological Sciences and Medical Sciences are geriatrics journals. Journal of Cachexia Sarcopenia and Muscle is a journal named sarcopenia. Nutrients and PLoS ONE are general journals.

**Table 1:** Top five most productive journals related to exercise interventions for sarcopenia.

**Rank**	**Publication**	**Journal**	**IF^a^**
1	225	Experimental Gerontology	4.253
2	191	Nutrients	6.706
3	121	Journal of Cachexia Sarcopenia and Muscle	12.063
4	115	PLoS ONE	3.752
5	85	Journals of Gerontology Series A-Biological Sciences and Medical Sciences	6.591

a IF, impact factor; IF in category according to Journal Citation Reports (2021).

**Figure 2:**
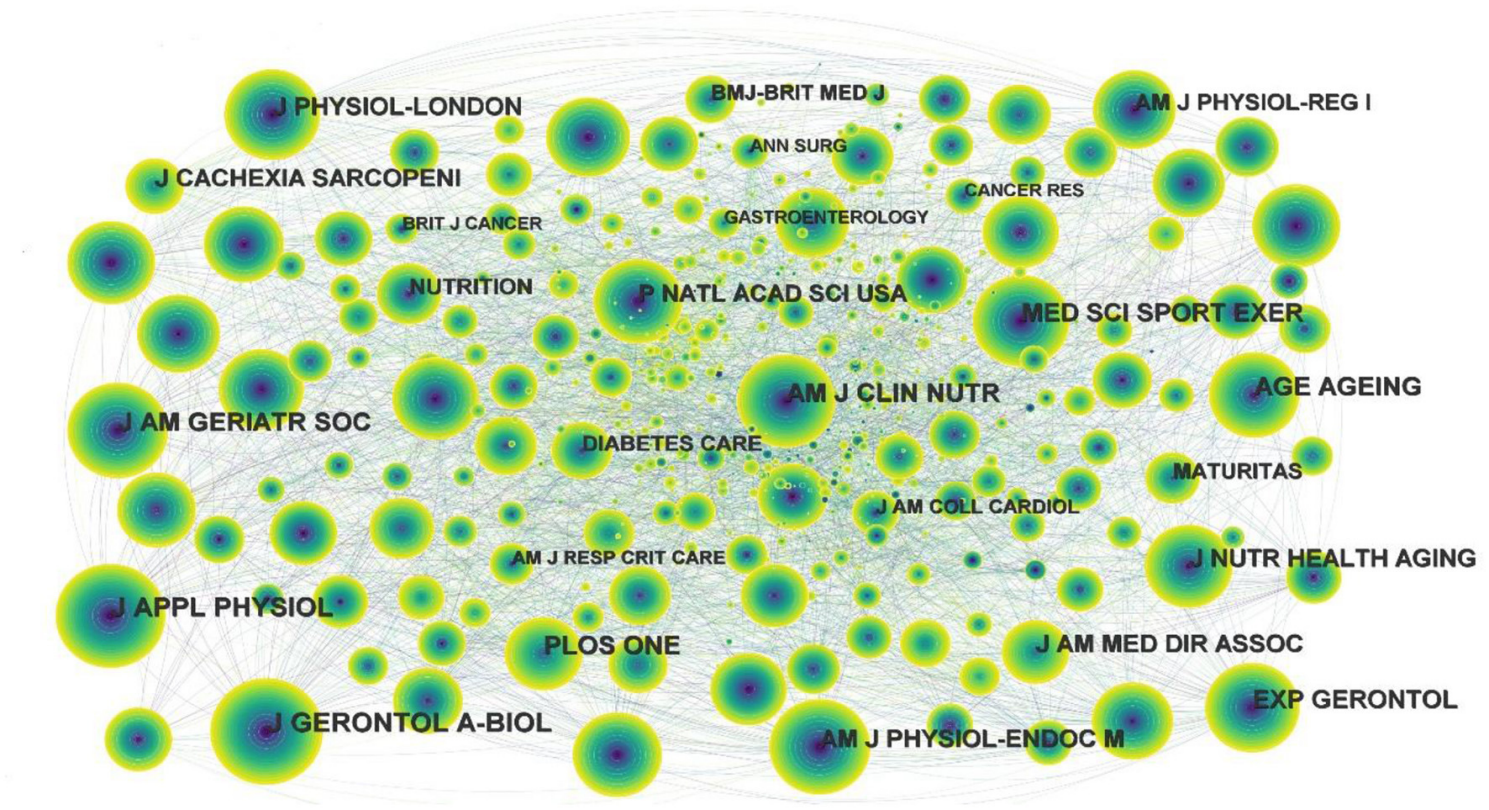
Map of cited journals for exercise interventions for sarcopenia from 2003 to 2022.

Moreover, the cited journal map was generated by CiteSpace (Figure 2), yielding 1,166 nodes and 11,707 links. The nodes in the map represent journals, and links between nodes represent co-citation relationships. As can be seen from the figure, there is no purple ring in these nodes, indicating that the centrality of these cited journals is not high, and ANNSURG with the highest centrality is only 0.05. The top 10 journals related to exercise interventions for sarcopenia are listed in Table 2.

**Table 2:** Top 10 cited journals related to exercise interventions for sarcopenia.

**Rank**	**Frequency**	**Cited journal**
1	3,433	J Gerontol A-Biol
2	3,059	J Appl Physiol
3	2,418	J Am Geriatr Soc
4	2,363	Age Aging
5	2,351	PLoS ONE
6	2,214	Am J Clin Nutr
7	1,966	Med Sci Sport Exer
8	1,931	Exp Gerontol
9	1,798	Am J Physiol-Endoc M
10	1,778	J Am Med Dir Assoc

### 3.3. Analysis of countries

**Figure 3:**
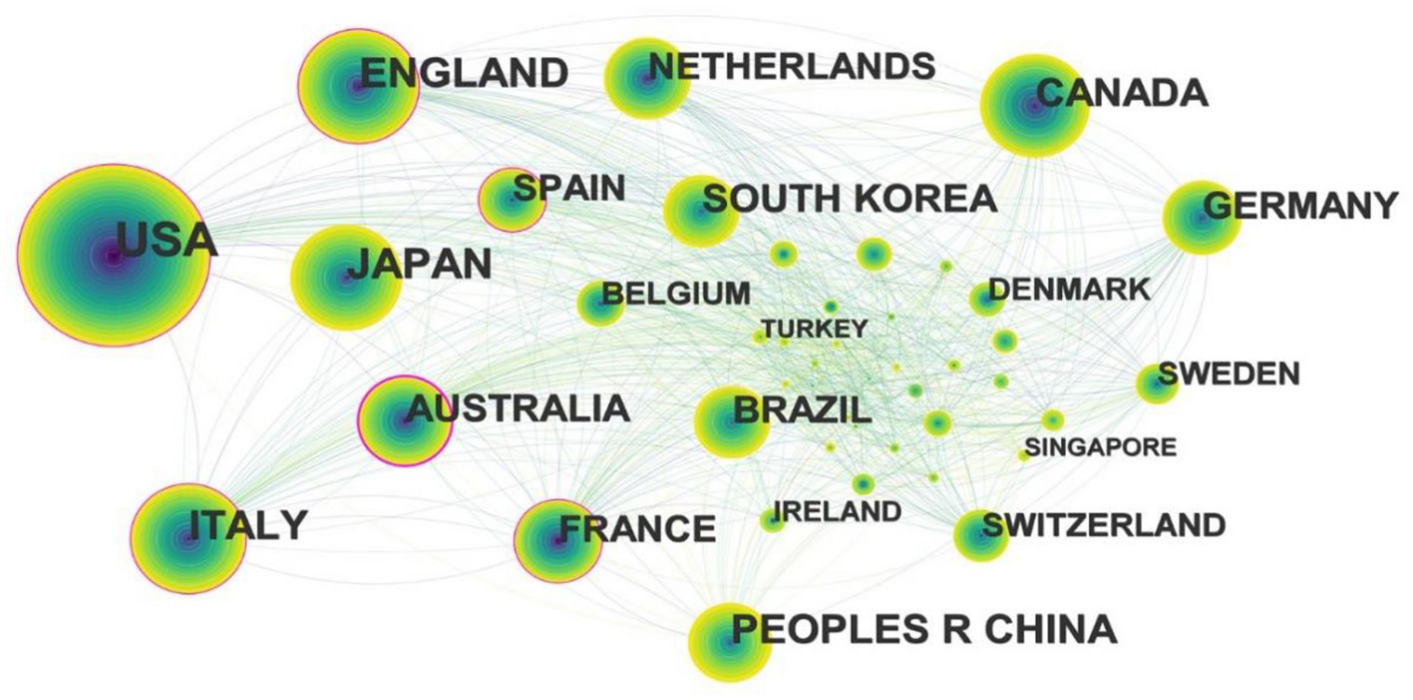
Map of countries for exercise interventions for sarcopenia from 2003 to 2022.

To understand which countries are most prominent in the field of exercise interventions for sarcopenia, we used CiteSpace to generate a country map (Figure 3). The 5,507 records were published in 84 countries, and the top five countries of publications are listed in Table 3. The United States of America was the country with the largest number of publications, accounting for about a third of the articles (1,619). Japan and England ranked second and third, respectively, followed by China and Italy.

**Table 3:** Top five countries in the number of publications related to exercise interventions for sarcopenia.

**Rank**	**Publications**	**Country**	**Earliest publication year**
1	1,619	USA	2003
2	612	Japan	2003
3	501	England	2004
4	489	Peoples R China	2005
5	465	Italy	2003

As shown in Figure 3, the nodes represent countries, and the purple rings indicated the centrality of the literature. The top five countries in centrality are Australia (0.27), Spain (0.2), England (0.19), USA (0.17), and France (0.13). The United States of America has an advantage in numbers, while Australia has an advantage in importance. In the following years, exercise interventions for sarcopenia has attracted increasing attention in Asian countries such as China and South Korea.

### 3.4. Distribution of institutions

**Figure 4:**
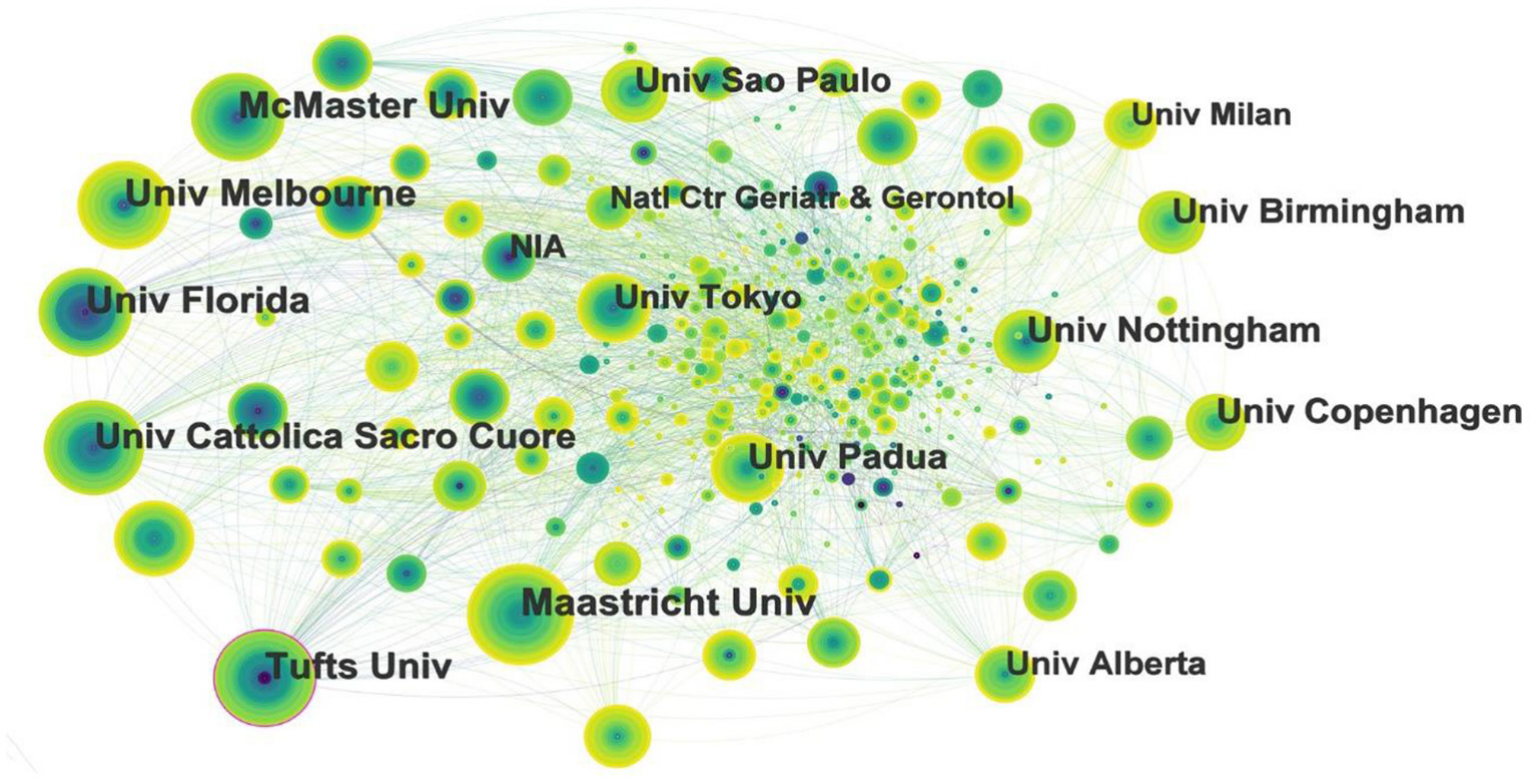
Map of institutions researching exercise interventions for sarcopenia from 2003 to 2022.

Among the 607 institutions with the highest attention in the field of exercise interventions for sarcopenia, the top 5 institutions are all comprehensive universities (Figure 4). They are Maastricht University, University of Florida, University of Melbourne, Tufts University, and McMaster University. The only two universities with centrality >1 were Tufts University (0.12) and the University of Florida (0.1). The number of publications and centrality analysis show that the main research institutions in this field are Tufts University and the University of Florida, which form the core of the complex cooperative network. Tufts previous research focused on sarcopenia diagnosis, nutrition and exercise interventions, body composition, and osteoporosis combined with sarcopenia. In recent years, however, Tufts has shifted its focus to the role of gut microbiota on muscle mass and strength in sarcopenia. The exercise interventions for sarcopenia research at the University of Florida mainly involves the combination with chronic diseases, inflammation, mitochondrial function, oxidative stress and so on.

### 3.5. Analysis of authors and cited authors

**Figure 5:**
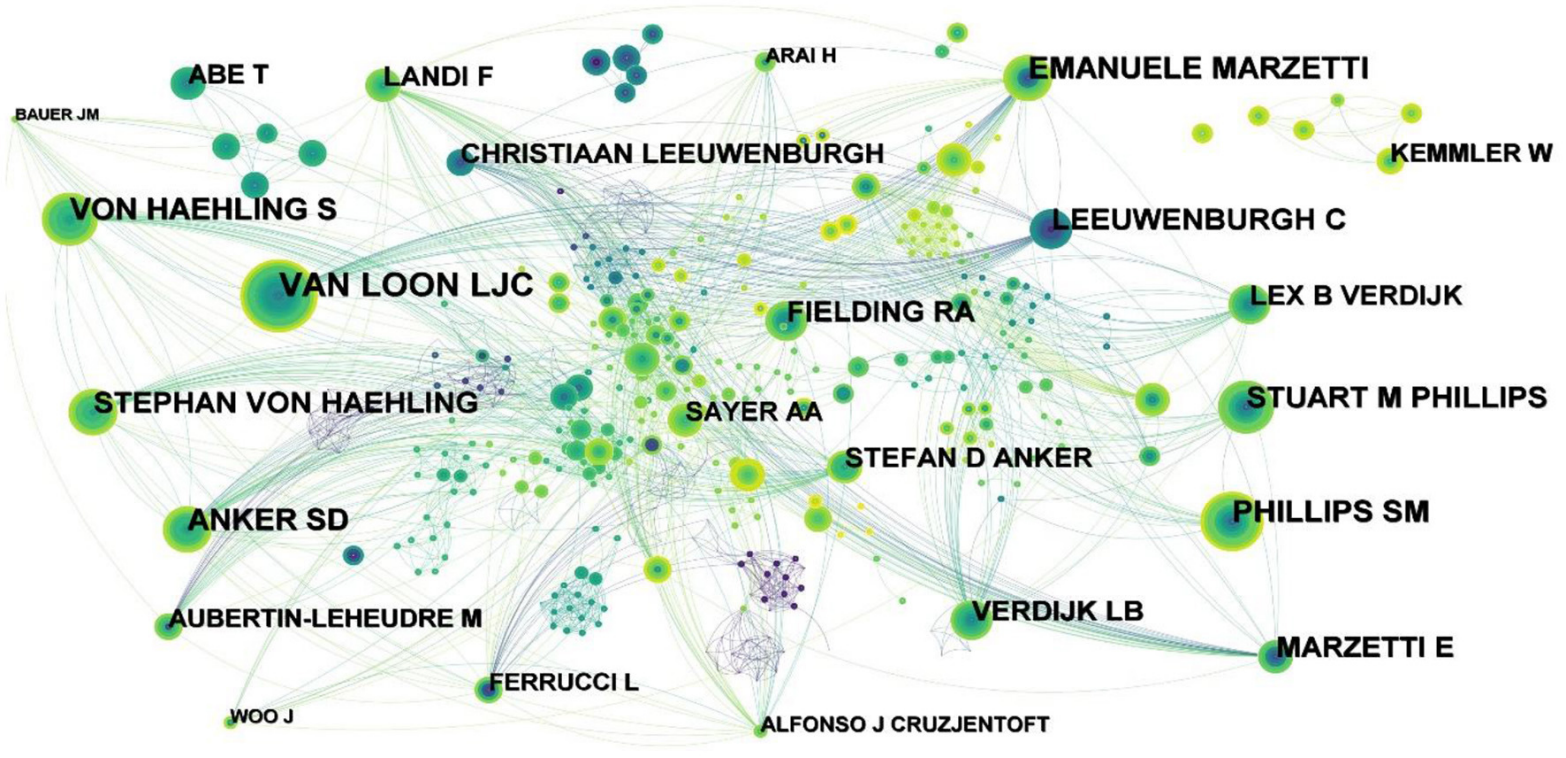
Map of authors related to exercise interventions for sarcopenia from 2003 to 2022.

The authors of the 5,507 records were analyzed, and 1,019 nodes and 3,517 links were obtained (Figure 5), indicating that 5,507 articles were published by 1,019 authors. The top five authors were VAN LOON LJC (53), PHILLIPS SM (37), EMANUELE MARZETTI (36), VON HAEHLING S (35), and ANKER SD (34). The research focus of VAN LOON LJC and PHILLIPS SM is similar, mainly focusing on the effects of exercise (10), protein supplementation on muscle protein synthesis (11–15) and muscle strength (16) in sarcopenia. PHILLIPS SM mainly focuses on nutritional supplements in support of resistance exercise to sarcopenia (17–20). EMANUELE MARZETTI not only focused on physical activity and exercise (21), but also analyzed biomarkers of frailty and sarcopenia to provide a reference for the diagnosis and detection of sarcopenia (22–24). VON HAEHLING S focuses on sarcopenia and cachexia caused by heart failure (25–28).

**Figure 6:**
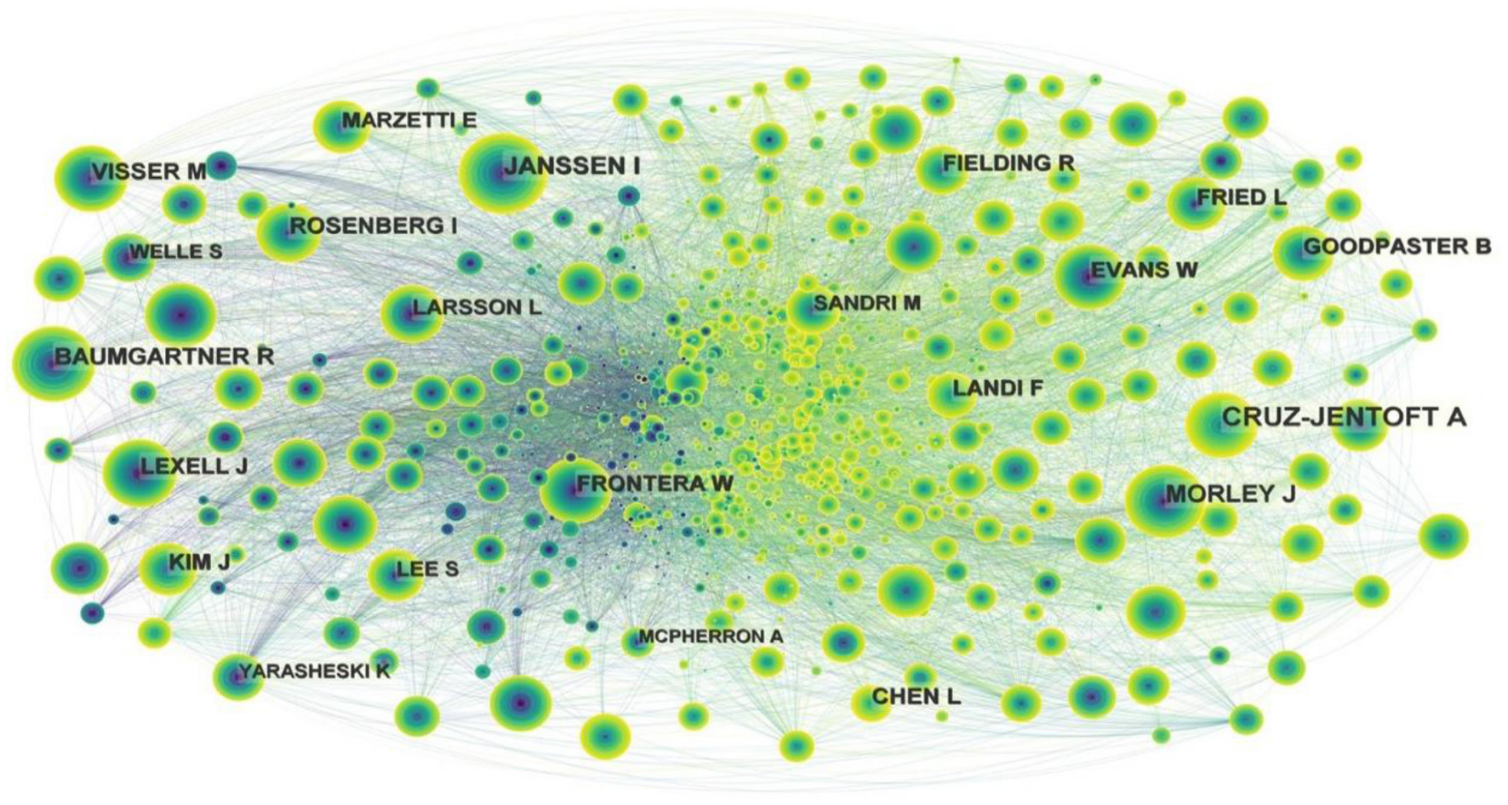
Map of cited authors related to exercise interventions for sarcopenia from 2003 to 2022.

The map of cited authors is shown in Figure 6. CRUZ-JENTOFT A had the highest citation counts (1,878), followed by JANSSEN I, MORLEY J, BAUMGARTNER R, and VISSER M (Table 4). Figure 6 shows that there is no purple ring, indicating that the centrality of cited authors are all < 0.1.

**Table 4:** Top five frequency and centrality of cited authors.

**Rank**	**Frequency**	**Author**	**Rank**	**Centrality**	**Author**
1	1,878	Cruz-Jentoft A	1	0.05	Fielding R
2	1,107	Janssen I	2	0.05	Yarasheski K
3	820	Morley J	3	0.05	Bhasin S
4	781	Baumgartner R	4	0.05	Mcpherron A
5	588	Visser M	5	0.04	Marzetti E

### 3.6. Analysis of cited references

**Figure 7:**
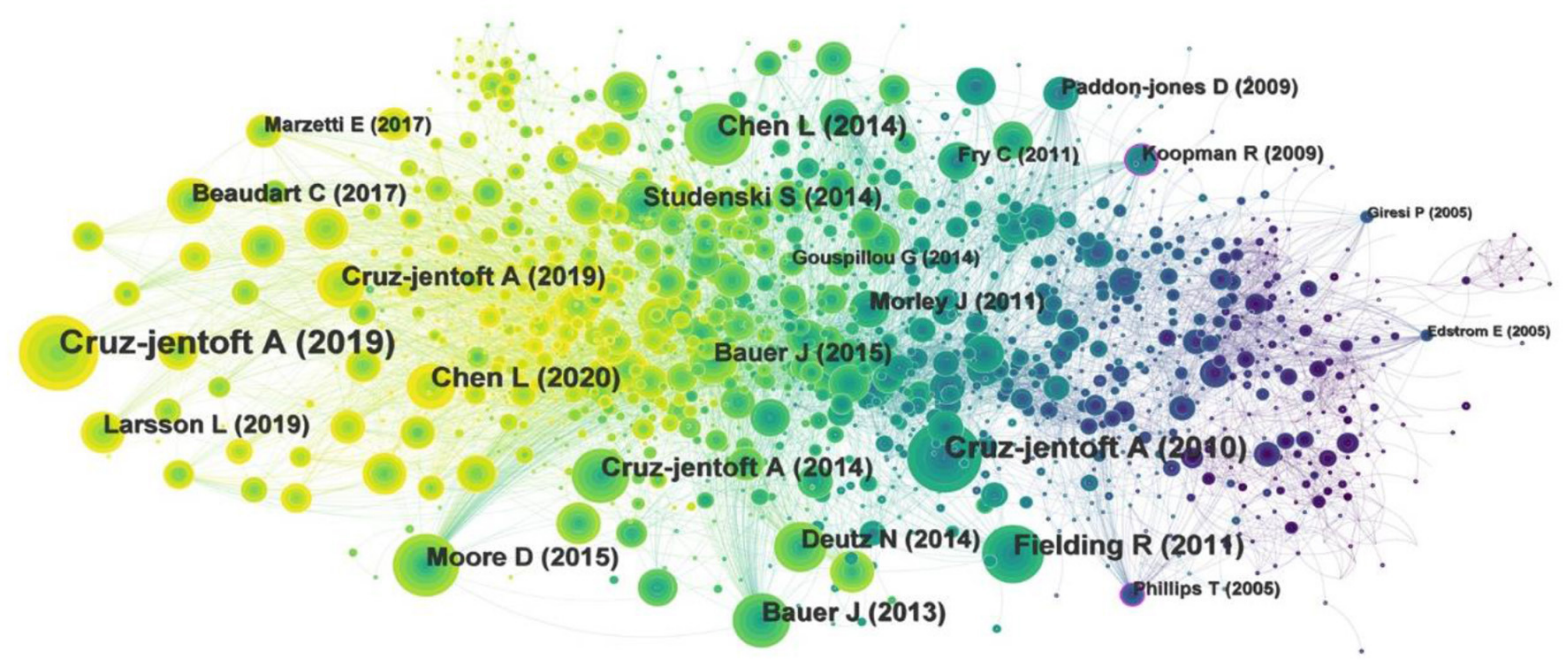
Map of cited references for exercise interventions for sarcopenia from 2003 to 2022.

Figure 7 is the cited reference co-citation map, yielding 1,618 nodes and 8,483 links. The two most frequently cited references are those published by CRUZ-JENTOFT A in 2019 and 2010, respectively (29, 30). These two articles detail the consensus on definition and diagnosis for sarcopenia of the European Working Group on Sarcopenia in Older People (EWGSOP) in 2019 and 2010. The third and fourth cited references detail the consensus on the diagnosis and treatment for sarcopenia of the Asian Working Group for Sarcopenia (AWGS), which was published by Liang-Kung Chen et al. in 2014 and 2020, respectively (1, 31). The fifth cited references was the expert consensus on the definition, prevalence, etiology and outcome for sarcopenia of the International Working Group on Sarcopenia published by Fielding R in 2011 (32). It can be seen that there is no unified definition and diagnostic criteria for sarcopenia at present, furthermore they are constantly updated. Different countries and regions may choose different diagnostic criteria. However, these working groups provide information on the definition, diagnosis and intervention of sarcopenia, which is a valuable reference for researchers engaged in sarcopenia.

The article published by Koopman R in 2009, with a centrality of 0.17, ranked first in cited references, which expounded the relationship between aging, exercise and muscle protein metabolism (33). This article pointed out that aging is accompanied by a steady loss of skeletal muscle mass and strength, age-related skeletal muscle mass loss due to interruptions of skeletal muscle protein turnover adjust to imbalances between muscle protein synthesis and degradation.

**Figure 8:**
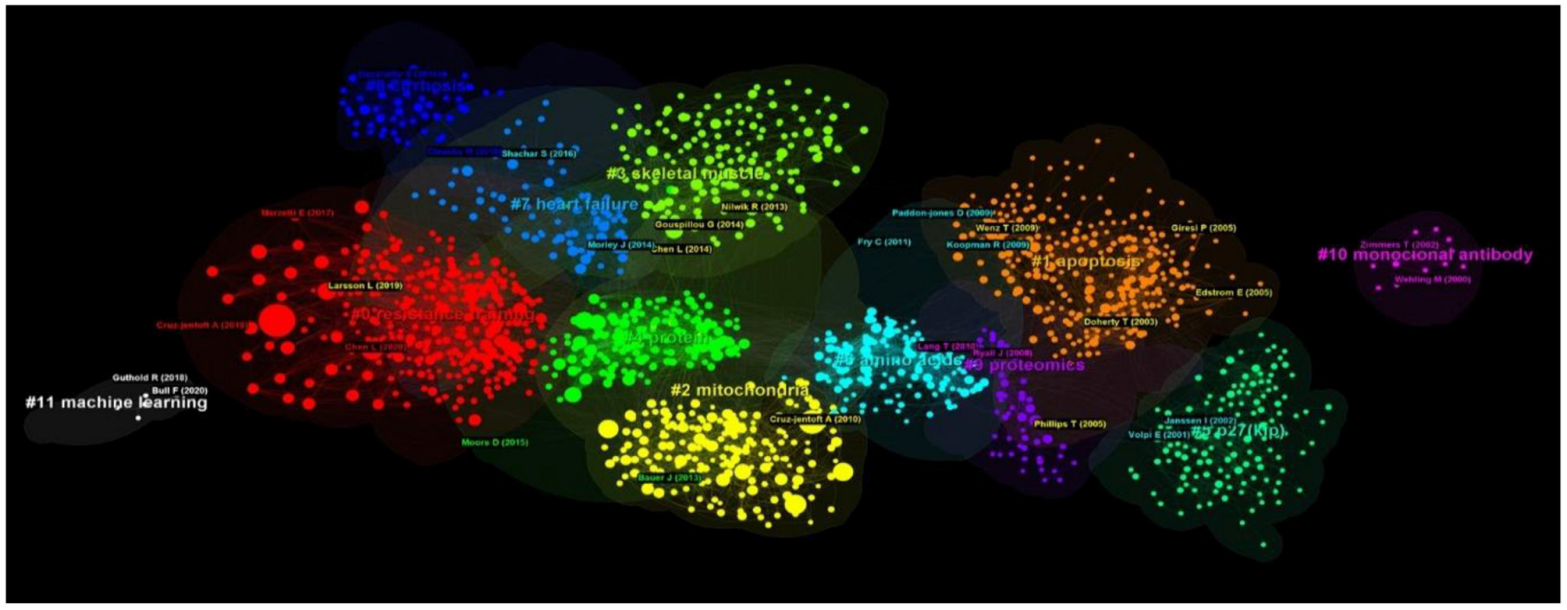
The clustering map of cited references related to exercise interventions for sarcopenia from 2003 to 2022.

We also performed cluster analysis on the cited references to clarify the topic and time distribution of these cited references (Figure 8). In this clustering map, the Q value is 0.6939 and S value is 0.8654, indicating than the clustering effect is valid and the credibility is high. A color region in the clustering map represents a topic. As can be seen from the color in the map, the topics of cited references focus on “resistance training,” “apoptosis, mitochondria,” “skeletal muscle,” “protein,” “amino acid,” “heart failure,” “cirrhosis,” “proteomics,” “monoclonal antibody,” and “machine learning.”

### 3.7. Analysis of keywords

**Figure 9:**
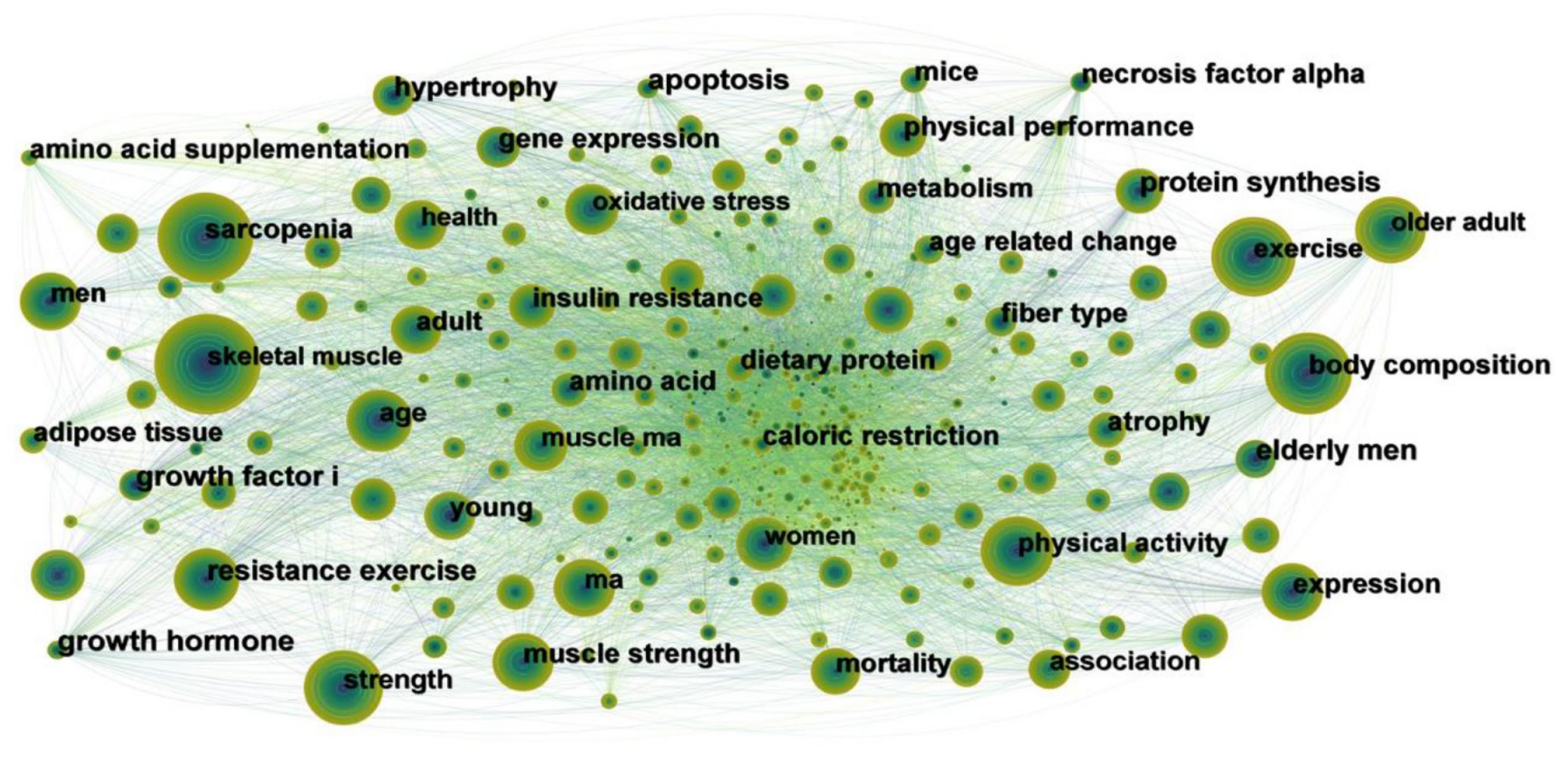
Map of keywords to exercise interventions for sarcopenia from 2003 to 2022.

It was considered that the increased frequency of keywords can reflect hot topics, and burst keywords which are cited frequently over a period of time could indicate frontier topics. We generated the network map of keywords, yielding 768 nodes and 9,176 links (Figure 9). A total of 768 research keywords were identified in the field of exercise interventions for sarcopenia, which reveals the hottest topics. According to the frequency and centrality of keywords (Table 5), it can be seen that the hot keywords are “sarcopenia,” “skeletal muscle,” “exercise,” “body composition,” “strength,” “older adult,” “physical activity,” “mass” and “resistance exercise,” while “resistance exercise” has a high frequency and centrality. The strength and mass of skeletal muscle are the focus of attention in sarcopenia. Therefore, the ultimate goal of any intervention is to improve the strength and mass of skeletal muscle, in order to improve the physical function of sarcopenia. Exercise is the most commonly used, easy to achieve, and effective method of sarcopenia rehabilitation. Resistance exercise is the most commonly used in the clinic, which has a significant effect on increasing the mass and strength of skeletal muscle.

**Table 5:** Top 10 frequency and centrality of keywords to exercise interventions for sarcopenia.

**Rank**	**Frequency**	**Keyword**	**Rank**	**Centrality**	**Keyword**
1	1,692	Sarcopenia	1	0.05	Growth factor
2	1,544	Skeletal muscle	2	0.04	Growth hormone
3	1,020	exercise	3	0.04	Apoptosis
4	898	Body composition	4	0.04	Caloric restriction
5	719	Strength	5	0.04	Necrosis factor alpha
6	685	Older adult	6	0.03	Resistance exercise
7	627	Physical activity	7	0.03	Insulin resistance
8	485	Mass	8	0.03	Young
9	474	Resistance exercise	9	0.03	Protein synthesis
10	433	Muscle strength	10	0.03	Age related change

**Figure 10:**
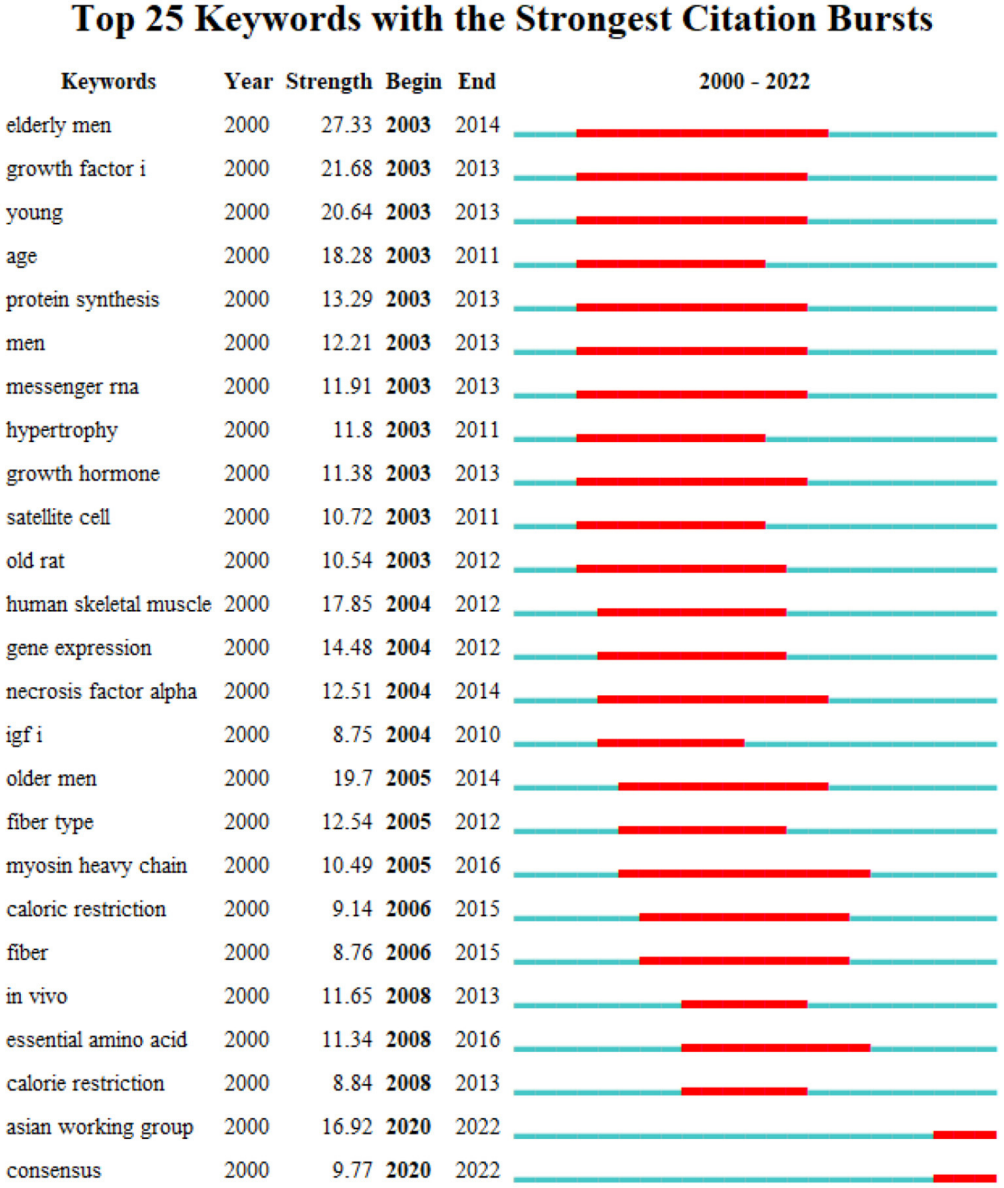
Top 25 keywords with the strongest citation burst.

The top 25 keywords with the strongest burst were obtained by time series burst detection of highly cited keywords (Figure 10). Among these keywords, “elderly men,” “young,” “age,” “men,” “old rat” are subjects of exercise rehabilitation studies for sarcopenia, “growth factors,” “protein synthesis,” “messenger rna,” “hypertrophy,” “growth hormone,” “satellite cell,” “necrosis factor alpha,” “necrosis factor alpha,” “igf i,” “myosin heavy chain,” “caloric restriction,” “essential amino acid” are the key research directions. The keyword “elderly men,” which emerged since 2003, showed the strongest citation burst of 27.33. The second most hot word was “growth factor” with strength of 21.68.

The most recent burst keywords were “Asian working group” and “consensus.” “Asian working group” means the Asian Working Group for Sarcopenia. In 2019, the Asian Working Group for Sarcopenia released the latest expert consensus on the diagnosis and treatment of sarcopenia (1), the highlight of which is that medical institutions at different levels should adopt different diagnostic strategies. The expert consensus also give detailed diagnosis and treatment methods for sarcopenia. At the same time, the diagnostic threshold is also updated, which is more operable.

**Figure 11:**
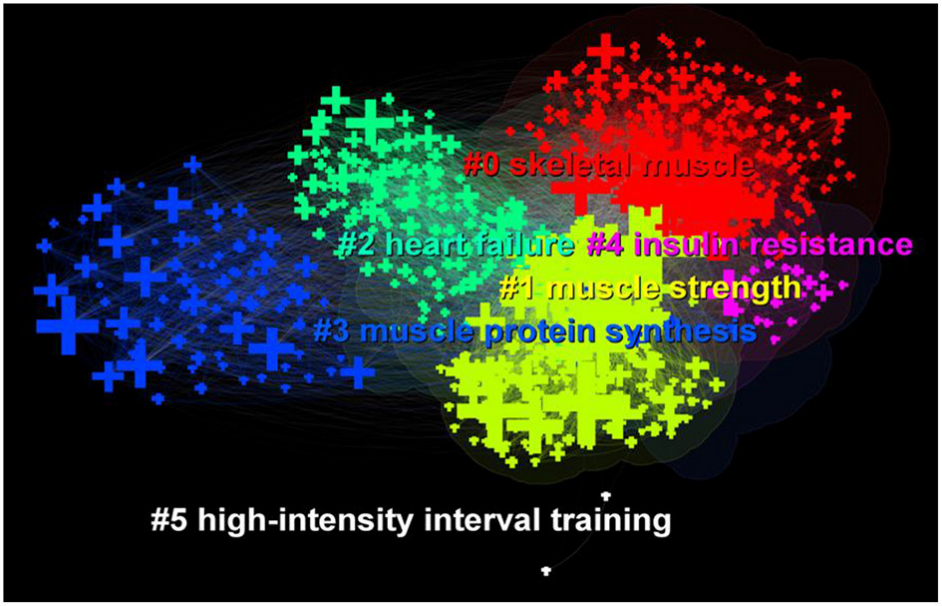
The clustering map of keywords related to exercise interventions for sarcopenia.

**Figure 12:**
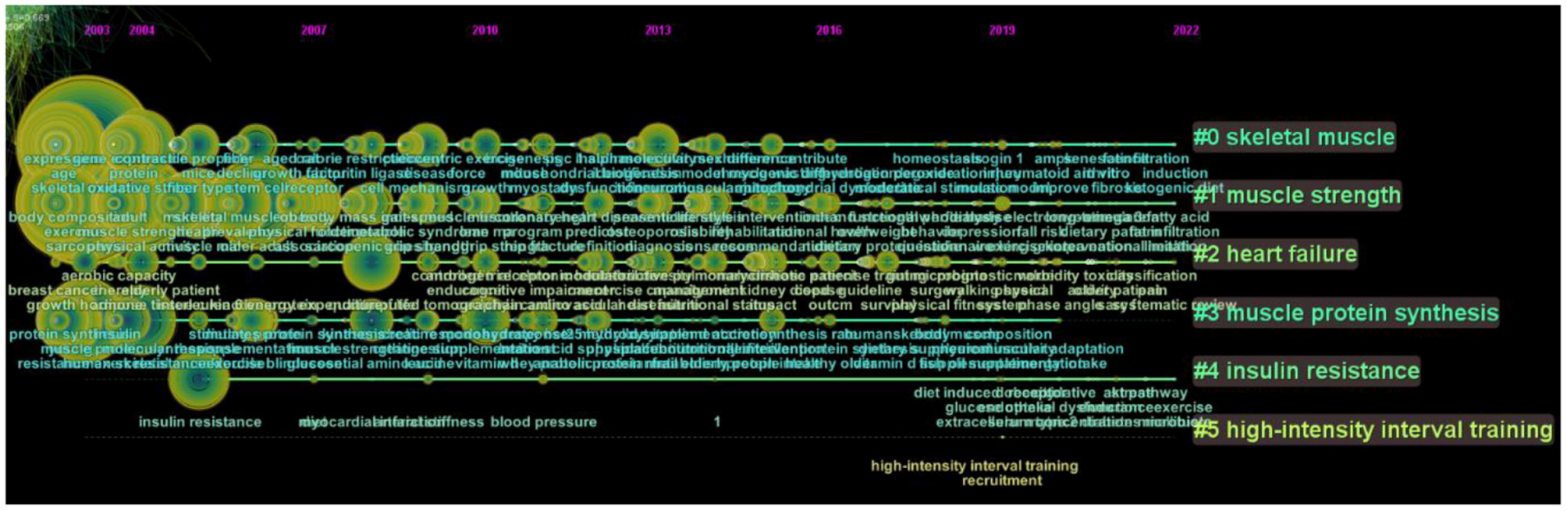
The timeline view of keywords related to exercise interventions for sarcopenia.

Cluster analysis and summary of these keywords can provide a more intuitive understanding of the current Research Topics related to exercise rehabilitation of sarcopenia (Figure 11). After clustering, the *Q*-value is 0.3397 and the *S*-value is 0.669, indicating that clustering is appropriate and meaningful. A total of six clusters were generated to reflect the hot trends, and they are “skeletal muscle,” “muscle strength,” “heart failure,” “muscle protein synthesis,” “insulin resistance” and “high-intensity interval training.” From the timeline view (Figure 12), “insulin resistance” and “high-intensity interval training” are the latest studies, while “skeletal muscle” and “muscle strength” appear earlier.

## 4. Discussion

### 4.1. General knowledge structure in exercise interventions for sarcopenia research

Exercise is a significant rehabilitation method for sarcopenia, which has received wide attention and is the key research direction of sarcopenia. Exercise rehabilitation has the advantages of easy operation and personalized exercise prescription. In this study, 5,507 records on exercise interventions for sarcopenia from January 2003 to July 2022 were retrieved from the Web of Science core collection. Based on the bibliometrics analysis of CiteSpace, we have clarified the spatial and temporal distribution and research hotspots of exercise interventions for sarcopenia. In the past 20 years, publications related to exercise interventions for sarcopenia have grown rapidly. In this study, the Journal of Experimental Gerontology published the most articles (225), and J GERONTOL A-BIOL was the biggest cited (3,433). The countries and institutions that carry out exercise interventions for sarcopenia research had relatively close cooperation. Generally, the United States of America, Australia, and some other European countries, with a high publication rate and centrality, all developed countries, were leading the field of exercise interventions for sarcopenia. The most productive institution was Maastricht University in the Netherlands. A total of 1,019 authors from different countries conducted studies on exercise interventions for sarcopenia. Of these authors, VAN LOON LJC was the most productive author, while CRUZ-JENTOFT A ranked first among cited authors. The cited reference with the highest centrality was a review, which focuses on the relationship between aging, exercise and muscle protein metabolism, published by Koopman R in 2009. Cluster analysis of the cited literature show that the most recently cited hot topics, respectively, were “resistance training,” “apoptosis, mitochondria,” “skeletal muscle,” “protein,” “amino acid,” “heart failure,” “cirrhosis,” “proteomics,” “monoclonal antibody,” and “machine learning.”

The hot keywords in the field of exercise interventions for sarcopenia are “sarcopenia,” “skeletal muscle,” “exercise,” “body composition,” “strength,” “older adult,” “physical activity,” “mass,” and “resistance exercise.” The keyword “elderly men” showed the strongest citation burst, and the second outbreak word was “growth factor.” A total of six clusters were formed, which, respectively, were “skeletal muscle,” “muscle strength,” “heart failure,” “muscle protein synthesis,” “insulin resistance” and “high-intensity interval training.” The keywords of “growth factors,” “protein synthesis,” “messenger rna,” “hypertrophy,” “growth hormone,” “satellite cell,” necrosis factor alpha,” “necrosis factor alpha,” “igf i,” “myosin heavy chain,” “caloric restriction,” “essential amino acid,” suggest the mechanisms or targets of exercise interventions for sarcopenia.

### 4.2. Main types of exercise for sarcopenia rehabilitation

Exercise therapy is an effective method to prevent and treat sarcopenia (34–39). The commonly used exercise methods include resistance exercise, aerobic exercise, combination of resistance and aerobic exercise, and other auxiliary exercise methods. The keyword related-information in this study shows that resistance exercise is the most commonly used.

#### 4.2.1. Resistance exercise

Resistance exercise as an effective way to enhance skeletal muscle protein synthesis, stimulate muscle hypertrophy and improve muscle strength, is the best-recommended exercise for sarcopenia (40–44). The resistance overcome during resistance exercise can be constant or incremental, which can be chosen according to the actual situation of the individuals. The types of exercises include weight lifting, seated leg lifts, static squats against the wall, stretching elastic bands, etc. For individuals with severe sarcopenia, rehabilitation equipment is often used to assist in training. The most recommended rehabilitation program for elderly participants with sarcopenia is at least 8 weeks, 3–4 times per week, actions at intensities of 40–60% of 1-repetition maximum, and for the untrained elderly, the exercise dose can be reduced to 2–3 times per week (45).

#### 4.2.2. Aerobic exercise

Although aerobic exercise cannot stimulate muscle hypertrophy, it can enhance muscle endurance level and muscle contraction capacity by effectively inhibiting the expression of apoptotic factors, improving cell mitochondrial quality and enhancing the activity of metabolic enzymes, thus maintaining muscle function and improving cardiorespiratory endurance (46). Aerobic exercise for about 30 min a day, 3 times a week, for more than 5 months can significantly improve the symptoms and prognosis of sarcopenia (37). Common aerobic exercises include walking, brisk walking, jogging, cycling, swimming, dancing, tai chi, setting-up exercise and some small-ball sports, etc. Participants can choose according to their own situation and interest. Aerobic exercise should start with low-intensity exercise (40% of maximum heart rate), lasting 5–10 min per day; medium intensity (50–60% of maximum heart rate), at least 10 min per time, 5 days per week; high intensity (>60% of maximum heart rate), at least 20–30 min per time, at least 3 days per week (47).

#### 4.2.3. Combination of resistance and aerobic exercise

Resistance exercise can significantly increase muscle mass and strength, but the effect of improving cardiopulmonary endurance is not significant. Aerobic exercise can effectively improve cardiopulmonary endurance, but the improvement of muscle function is limited. Furthermore, the single exercise mode is easy to produce muscle fatigue, lack of fun, and it is difficult for participants to adhere for a long time. Therefore, multi-component exercise is often used clinically to intervene in participants with sarcopenia, such as moderate resistance training, aerobic + resistance training, resistance + balance + gait training and resistance training + outdoor activities, etc. The multi-exercise approach is not only effective, but also more stimulating to participants' interest in exercise (44, 48).

#### 4.2.4. Other auxiliary exercise

Participants with sarcopenia have a gradual loss of balance ability and postural control disorders, which become important factors leading to fall and poor physical flexibility (49, 50). Therefore, it is recommended that older adults perform balance training, such as cross-pacing and tai chi (51), more than three times a week as a way to reduce the risk of falls. At the same time, a cumulative total of at least 2 weeks of flexibility training should be conducted each month, with a moderate to light dose of 10 min each time, including the neck, shoulders, kinesiology, knees and other joint parts (52). For elderly people who are constrained by physical conditions or unwilling to carry out dynamic exercise training, whole body vibration therapy can be used to stimulate the muscles, which can also achieve desired results (53–55). In addition, participants with sarcopenia should try to reduce sedentary and bed-ridden in daily life, and should increase the frequency of daily activities, such as gardening, traveling, housework, shopping, climbing stairs, etc., which can play a role in exercising limb strength.

In this study, six clusters were obtained after cluster analysis of keywords. One of the keywords was high-intensity interval training (HIIT), which has attracted much attention in recent years. Multimodal HIIT has a combination of aerobic and anaerobic performance effects, and has significant effects on improving muscle strength and muscle endurance. HIIT also produce beneficial effects on cardiorespiratory fitness, physical fitness, muscle strength, cardiac contractile function, mitochondrial citrate synthase activity, and lowering blood triglyceride and glucose levels in older adults (56). However, it is worth mentioning that HIIT, as a high-intensity exercise mode, should only be performed by participants with sarcopenia after a systematic physical assessment to determine the appropriate exercise dose. Currently, there are no studies directly targeting participants with sarcopenia for high-intensity interval training due to safety reasons (57).

In addition, the results of several studies confirm that exercise combined with nutritional supplementation is the best choice for the rehabilitation of sarcopenia. Supplementation with protein, amino acids, vitamin D and other nutrients along with exercise intervention can promote muscle protein synthesis, muscle cell proliferation and differentiation, significantly delaying muscle aging (19, 58–61).

### 4.3. Future trends

Timeline view analysis and burst detection methods were used to identify cutting-edge content and reveal future trends. From Figure 12, we found that heart failure, muscle protein synthesis, insulin resistance, and high-intensity interval training are the hot spots of research in recent years. With the emergence of diversified exercises and their good results in the rehabilitation of sarcopenia, researchers are increasingly concerned about the mechanism of action and safety of exercise for sarcopenia. Sarcopenia was a common condition in participants with heart failure, and heart failure can induce sarcopenia. Therefore, early detection of sarcopenia and appropriate interventions are important in heart failure (62–64). The aim of exercise interventions for sarcopenia is to improve muscle mass and strength. The analysis of timeline view revealed that protein synthesis and insulin resistance are closely related to muscle mass and strength, which provides a direction to study the mechanism of exercise interventions for sarcopenia.

### 4.4. Strengths and limitations

This study summarized the hotspots and research trends of exercise for sarcopenia, and provided a meaningful reference for the research in this field. However, this study also has some limitations. Firstly, we only analyzed data from the Web of Science core collection. Searches are not selected in PubMed, Scopus, or other databases. Second, the literature contains papers in English only; the status of research on exercise interventions for sarcopenia in other language nations is not possible to determine. Lastly, the number of citations and centrality of articles may vary if the search is conducted at different time periods. Therefore, this study only represents the hotspots in the last 20 years.

## 5. Conclusion

This study uses the visualization software CiteSpace to identify potential collaborators and partner institutions, hot topics and new perspectives on research frontiers in the research of exercise interventions for sarcopenia. Bibliometric analysis of the literature shows that exercise, as the most significant intervention, remains the most cutting-edge and critical component in the rehabilitation of sarcopenia. Looking for scientific, effective and safe exercise prescriptions will be the focus of future research. In conclusion, our study provided a comprehensive landscape of the development and identified the key features of exercise interventions for sarcopenia over the past 20 years. This may provide a direction for the exploration and development of exercise interventions for sarcopenia.

## Author contributions

QX contributed to conception and design of the study and obtaining funding for the study, and wrote the first draft of the manuscript. YH and JZ contributed to data collection and analysis. WL and JT contributed to manuscript revision. All authors contributed to manuscript revision, read, and approved the submitted version.
